# Effectiveness of a community-based multifaceted fall-prevention intervention in active and independent older Chinese adults

**DOI:** 10.1136/ip.2008.020420

**Published:** 2009-07-27

**Authors:** Q H Xia, Y Jiang, C J Niu, C X Tang, Z L Xia

**Affiliations:** 1 Changning Center for Disease Control and Prevention, Shanghai, China; 2 Fundan University, Shanghai, China

## Abstract

**Objective:**

To evaluate the effectiveness of an 18-month multifaceted intervention designed to reduce the incidence of falls in community-living older adults in China.

**Methods:**

A population-based community trial evaluated by before-and-after cross-sectional surveys. Four residential communities were randomised to either a multifaceted intervention or a control condition. Baseline information was collected from a sample of older adults in each community. A 1-year annual fall rate was calculated after an 18-month comprehensive intervention.

**Results:**

After intervention, 7.19% of the intervention community sample reported falls, compared with 17.86% of the control community sample (p<0.000). The annual fall rate decreased by 10.52% in the intervention communities, whereas the difference in control communities was not statistically significant.

**Conclusions:**

Multifaceted interventions in community settings may be useful in preventing falls among older people, and can be applied in similar settings in China.

The population of older adults is rapidly increasing in China. According to the Shanghai Public Security Bureau, there were approximately 2.8 million adults aged 60 or older in Shanghai at the end of 2006, accounting for 20.1% of the city’s population. Falls and fall-related injuries are one of the most common health problems among older adults. Although statistics from different populations vary, 15.9% of older adults reported falling over 3 months in the USA,^1^ and 28.5% of older people in Turkey were found to have fallen within 1 year.^2^ The annual incidence of falls in Chinese Hong Kong is 26.4%.^3^

Fall injuries among older people have adverse effects on quality of life and can be burdensome to families and society. Non-fatal fall injuries are associated with considerable morbidity including decreased functioning, loss of independence and significant use of healthcare services.^4^^–^7 One survey conducted in China suggested that 73.4% of these falls result in injuries.^8^ In a US sample, 31.3% of those who fell sustained an injury that resulted in a doctor visit or restricted activity for at least 1 day.^1^ It was calculated that fallers use US$71 million more public healthcare dollars than non-fallers did annually in Hong Kong.^3^

Risk factors for falls are multiple.^9^ ^10^ A number of previous studies have indicated that falls in older adults result from interactions between internal risk factors (chronic diseases, visual problems) and environmental factors (including unsafe footwear, inadequate lighting and prescription drug use).^11^^–^13 Several multifaceted fall-prevention interventions have been shown to be effective.^14^^–^16

The community is a major component of an older person’s daily life in Shanghai. About 61.7% of falls among the older population occur in a community setting, including their homes.^10^ As a result, the community is a major target location for fall prevention, but there have only been a few studies addressing the effectiveness of a community-based intervention for falls in this age group in Hong Kong,^17^ and no such studies reported from the Chinese mainland. Interventions efficacious in other settings may not be effective in Chinese communities because of differences in the concept and construction of community groupings. In addition, the idea that injury is preventable is often an unfamiliar concept among medical professionals and community members alike. The aim of this study is to explore the feasibility and efficacy of a multifaceted fall intervention in communities of older urban Chinese.

## METHODS

### Background information

In Shanghai, older adults spend most of their time within a community setting, particularly after retirement. The organisational structure of local authorities is as follows: every district government comprises street governments, which in turn comprise residential committees. Every committee represents a community of 1000–2000 families.

### Study subjects

Four residential communities in Shanghai were selected, and within each community, those aged 60 years and older were included in this study. These communities were comparable in economy, size and other aspects. Each community had about 900 older adults. Those unable to walk without the assistance of another person, unable to answer the interview questions, or living in a nursing home were excluded. The four communities were randomly allocated to either an intervention group or control group. Intervention group members were eligible to receive all intervention measures. The follow-up period lasted from January 2006 to September 2007. Subjects from each group were randomly selected from a list of all residents before (n = 2310) and after (n = 1422) intervention to complete evaluation measures, and the response rate was 96.3% and 94.9%, respectively.

We received oral informed consent before implementing the programme and again at the time of administering the questionnaire.

### Definition of a fall

For the purpose of this study, a fall was defined as an “unexpected or unintentional fall to the ground or to a surface lower than the center of gravity, excluding sudden occurrence of stroke, paralysis or epilepsy.”^18^

### Multifaceted intervention design

The intervention framework include behavioural and environmental components, individual and group interventions, and interventions directed at older people and their carers (see table 1). Behavioural interventions included an education programme, brochure distribution, poster exhibition and healthcare consultation. Environmental interventions included improving indoor and community safety through hazard assessment and hazard elimination. Every participant in the intervention group had access to all these interventions.

**Table 1:** Main intervention measures

Intervention	Description	Coverage*(%)	Frequency
Education programme (community lecture)	Provide guidance on fall-related aspects of diet, dwelling, movement, exercise and medicine use. Topics included: selecting suitable clothing and shoes using crutches properly improving balance and gait obtaining help after falling common risk factors for falls	100	Once every 2 months
Healthcare consultation	Consult in fall prevention and related health problems; give advice	34	Once every 2 months
Poster exhibition and DVD display	Poster board with contents similar to community lectures.	100	Throughout the intervention period
In-home hazard assessment	Assess and reduce the risk of falling in the home. Indoor fall risk factors included: poor lighting slippery floor surfaces objects in walkway unstable furniture loose rugs shelves or cupboards too high or too low lack of safety rails in the toilet and bathroom stairs too steep	95.2	Twice a year
Brochure distribution	Brochures with the same contents as community lectures were delivered to all people in the intervention group	100	Throughout the intervention period
Modify community settings	Periodically assess and modify risk factors in the community. The risk factors included: uneven pavement holes in lawn obstacle on road lack of handrails	100	Twice a week

*Coverage represents the proportion of the eligible subjects in the intervention group who have actually taken part in each activity.

Before the intervention was conducted, a multidisciplinary group was established, including the local centre for disease control and prevention (CDC), representatives from the street government, the community health centre (CHC), community committees, landowners within the community, and volunteers.

The intervention was led by the CDC. Healthcare professionals from the CHC collected fall incidence information and carried out in-home hazard assessments. Street governments provided policy support to guarantee sustained fall-prevention efforts through follow-up and cooperation of landowners in fall risk factor elimination. Community committees assisted in organisation of participants. Trained volunteers were responsible for providing education on exercise techniques, as well as collecting from community members “golden ideas on prevention of falls”, or add these to the education programme. This multidisciplinary group provided help and advice to subjects in the intervention group through a number of intervention programmes.

### Data analysis

A baseline investigation was conducted before intervention. Trained interviewers administered questionnaires to samples selected randomly from each group. The key measures were: self-reported fall experience and information on birth date, sex, education, marital status, chronic disease conditions, activities of daily living,^19^ ^20^ knowledge, attitudes and behaviours relevant to fall risk and prevention. The same questionnaire was used at both baseline and the 18-month follow-up.

EPIDATA V3.0 was used for data entry, and statistical analyses were conducted using SPSS V11.5. The χ^2^ test was used to compare annual fall rate and characteristics of participants before and after intervention. All p values are two-tailed at the significant level of α = 0.05.

## RESULTS

The baseline characteristics of the subjects, including demographic information, health status, knowledge about and attitudes towards preventing falls were similar in control and intervention community samples (p>0.05) (table 2), although a higher proportion of the control group had taken preventive measures such as wearing non-slippery shoes (p = 0.03).

**Table 2:** Characteristics of participants in control group and intervention group

	Control (n = 994)	Intervention (n = 1316)	p Value
Mean (SD) age (years)	71.8 (6.9)	72.3 (7.7)	0.112
Female	505 (50.8)	708 (53.8)	0.154
ADL score			0.686
Good	75 (67.9)	900 (68.4)	
Slight impairment	78 (7.8)	113 (8.6)	
Obvious dysfunction	41 (24.2)	303 (23.0)	
Self-reported health status			0.642
Good	25 (12.6)	183 (13.9)	
Normal	681 (68.5)	885 (67.2)	
Poor	88 (18.9)	248 (18.8)	
Believe older adults are susceptible to falls	537 (54.0)	740 (56.2)	0.291
Believe falls are preventable	514 (51.7)	644 (48.9)	0.368
Take fall-prevention measures	228 (22.9)	254 (19.3)	0.033

Unless otherwise indicated, values are number (%).

ADL, activities of daily living.

Our intervention measures were welcomed by all residents. However, 4.8% of all subjects in the intervention community sample refused in-home visits by healthcare professionals. To these people, we gave recommendations on evaluating and eliminating the risk factors in their homes. Because they had access to other intervention programmes, their outcomes are included with their study group for evaluation.

Table 3 shows that the incidence of falls and the rate of multiple falls decreased by 10.5% (χ^2^ = 42.893, p = 0.00) and 6.1% (χ^2^ = 37.270, p = 0.00), respectively, in the intervention community sample, while there was only subtle change in control community samples. The incidence/rate ratio for falling in the intervention group, as compared with the control group, was 0.356 (95% CI 0.253 to 0.501). After 18 months of intervention, there was a significant difference in the proportion of fall-related injury and facture between the intervention group and the control group. The incidence/rate ratios for fall-related injury and fracture were 0.32 (95% CI 0.23 to 0.44) and 0.43 (95% CI 0.22 to 0.77).

**Table 3:** Annual fall rate before and after intervention

Number of falls in previous 12 months	Before intervention (%)		After intervention (%)	
	Control (n = 994)	Intervention (n = 1316)	Control (n = 699)	Intervention (n = 723)
1	18.3	17.7	17.9	7.2*
⩾2	9.3	7.2	6.0	1.1*
Fall-related injury	–	–	17.6	6.9*
Fall-related fracture	–	–	4.3	2.4*

*Significant difference (p<0.05) between intervention group and control group after intervention.

Knowledge, attitudes and behaviours related to fall prevention were assessed for subjects of the intervention and control communities at baseline. Table 4 shows that more than half of the participants were aware of falls, and the percentage of people who believed that falls can be prevented was relatively high in both groups. After intervention, there was a significant increase in the percentage of people who believed that falls were preventable (72.8% vs 48.9%, p<0.05) and take measures to prevent falls in the intervention communities (86.6% vs 19.3%, p<0.05). However, there was no difference in the use of walking aids between the two groups after intervention (p>0.05). Table 5 presents the specific measures adopted by older people to prevent falls.

**Table 4:** Fall-related knowledge, attitudes and practices of the subjects in both groups before and after intervention

Item	Before intervention (%)		After intervention (%)	
	Control (n = 994)	Intervention (n = 1316)	Control (n = 699)	Intervention (n = 723)
Aware of falls in older adults	54.0	56.2	50.0	56.3*
Use walking aid	10.8	12.9	13.3	15.5
Believe falls can be prevented	49.1	48.9	57.3	72.8*
Take measures to prevent falls	22.9	19.3	34.3	86.6*

*Significant difference (p<0.05) between intervention group and control group after intervention.

**Table 5:** Rate of taking preventive measures after intervention in the control and intervention groups

Preventive measure	Control (n = 699)	Intervention (n = 723)
Choose suitable clothes and non-slippery shoes	5.0	32.1*
Be careful while moving about at home	22.9	53.1*
Be careful while walking outdoors	43.1	67.2*
Do exercises to improve balance and strength	19.3	31.7*
Take medicine according to instructions	5.00	21.58*
Keep in good spirits	5.57	13.14*

Values are percentages.

*Significant difference (p<0.05) between intervention group and control group after intervention.

Compared with those in the control communities, more subjects in the intervention communities took measures to prevent falls after intervention (p<0.05). The prevention method of the subjects in the control group was mainly to “be careful”. There was an increase in the use of suitable, safe, clothing. In addition, an increase in exercise was also observed in the intervention group (table 5).

When falls were stratified according to age, we found that the multifaceted intervention was not as effective in the 60–69 age group as in those older than 70 years. There was no significant difference between the intervention group and control group when the age was <70 (p>0.05) (table 6). A similar pattern of changes in knowledge, attitudes and practice was observed in the two groups (table 7).

**Table 6:** Age-specific fall rate before and after intervention in the control and intervention groups

Age group	Before intervention (%)		After intervention (%)	
	Control (n = 994)	Intervention (n = 1316)	Control (n = 699)	Intervention (n = 723)
Fell once in last 12 months				
>60	15.5	13.3	12.6	7.6
>70	19.3	18.4	21.4	6.1*
>80	22.7	25.1	17.5	9.2*
Fell ⩾2 times in last 12 months				
>60	7.8	4.5	4.1	0.9*
>70	9.1	7.8	6.2	1.2*
>80	15.1	11.3	7.3	1.2*

*Significant difference (p<0.05) between intervention group and control group after intervention.

**Table 7:** Age-specific change in knowledge, attitudes and practices in older people after intervention

Age group	Before intervention (%)		After intervention (%)	
	Control (n = 994)	Intervention (n = 1316)	Control (n = 699)	Intervention (n = 723)
Believe that fall can be prevented				
>60	56.0	58.2	64.4	68.4
>70	46.1	46.2	52.2	66.9*
>80	41.2	36.8	58.4	90.9*
Take preventive measures				
>60	22.2	21.4	59.9	84.9*
>70	21.8	16.9	56.0	83.3*
>80	30.3	20.9	40.9	95.7*

*Significant difference (p<0.05) between intervention group and control group after intervention.

## DISCUSSION

This study suggested an association between reduction in fall incidence in older adults and a multifaceted community intervention. Similar results were found by some earlier studies.^21^ After intervention, the number of risk factors for falls was reduced in both people’s homes and community settings, and more people changed their practices and attitudes towards fall prevention. Most people paid close attention to avoiding falls while walking, but the percentage who paid attention to risk factors such as diet, medication and disease was still low. Education had little effect in promoting habits such as the use of walking aids, which are perhaps more dependent on health condition.

The effectiveness of multifaceted interventions among older people differed by age group, with greatest efficacy in the oldest participants. As people age, with muscle strength and physical function decreasing, they may pay more attention to their health, and are more willing to accept information on how to promote beneficial habits.

The effectiveness of multifaceted interventions has been studied, but not all components of multiple intervention programmes are likely to be effective.^22^ Some trials using a single intervention, such as balance and strength retaining,^23^ withdrawal of psychotropic medicine,^24^ or improving vision, showed little effect.^25^ In our intervention, we did not study the effectiveness of individual intervention components, and further research is necessary. In addition, the cost-effectiveness of fall prevention has not been established, and careful economic modelling in the context of a local healthcare system is needed.

Our study also identified a feasible model of collaboration, which could be applied in research and disease prevention at the community level. A multidisciplinary group, comprising the local CDC, street authorities, the CHC and residential committees was found to be a viable network for carrying out sustained community intervention. If this intervention model were applied in communities across urban areas in China, we might decrease the incidence of falls appreciably, and improve the health and quality of life of older adults.

### Study limitations

As it was a community intervention, all active older adults of the intervention group living in communities were exposed to the intervention, but to evaluate the effectiveness of the intervention, only a random sample was interviewed. The individuals in this sample differed from baseline to follow-up. Our analysis was thus a repeated cross-sectional study rather than a longitudinal cohort study. The outcome we measured was self-reported fall episodes; as a result, fatal injuries were not assessed. Our findings may thus underestimate the impact of fall-related injuries. Finally, we note that the present findings cannot be generalised to older people living in nursing homes or hospital settings.

**What is already known on this topic**

- Multifaceted intervention may reduce fall-related risk factors and falls among older people in community settings.
- No such programmes have been evaluated in the unique community context of urban China.

**What this study adds**

- We describe a feasible model of community collaboration to prevent falls that can be applied in research and disease prevention.
- A multifaceted intervention in a Chinese community setting was associated with fewer falls among older residents.
- These interventions seemed to be most effective in the oldest study cohorts.
